# Chinese undergraduate students’ motivation to learn Korean as a LOTE

**DOI:** 10.3389/fpsyg.2023.1063363

**Published:** 2023-07-04

**Authors:** Lin Su, Jaewoo Shim, Heechul Lee

**Affiliations:** Department of English Education, College of Education, Jeonbuk National University, Jeonju, South Korea

**Keywords:** L2MSS, motivation, Korean as a LOTE, future self-guides, Chinese undergraduate students

## Abstract

As rates of multilingualism increase, interest in the field of Languages Other Than English (LOTEs) has been growing over the last few years. This study investigated the motivation held by Chinese undergraduate students for learning Korean as a LOTE using Dörnyei’s L2 Motivational Self System (L2MSS). In total, 123 subjects responded to the 6-point Likert scale measuring their Korean learning motivation. The collected data were analyzed using SPSS 22. Logistic regression was applied for identifying variables that distinguished the first-year from the second-year learners of Korean, while canonical correlation analysis was used to examine the correlation between two sets of variables, the first set of dependent variables of the *ideal L2 self* and the *ought-to L2 self*, and the second set of independent variables of *family influence*, *instrumentality promotion*, *instrumentality prevention*, *attitude to learning Korean*, *cultural interest*, *attitude toward community* and *integrativeness*. Results showed that variables of *family influence*, *cultural interest*, and *attitude to learning Korean* were statistically significant in distinguishing the first-year from the second-year learners in terms of affective variables. In addition, canonical analysis showed that the dependent variable set of the *ideal L2 self* and the *ought-to L2 self* together shared nearly 69% variance with the independent variable set, indicating that the *ideal L2 self* and the *ought-to L2 self* together were highly related with these affective variables in the independent variable set. The findings of the current study suggest that more creative Korean language learning activities be adopted to help sustain the high levels of affect among Korean language learners.

## Introduction

With the increase in the number of people who speak and learn multiple languages, motivation to learn additional languages has been receiving great attention from researchers (Ushioda, 2011, 2017; Douglas Fir Group, 2016; Lanvers et al., 2016; Henry, 2017; Siridetkoon and Dewaele, 2018; Dörnyei, 2019). Nonetheless, literature in this field revealed that nearly 73% of the empirical studies focused on Global English learning, leaving Languages Other Than English (LOTEs) understudied (Boo et al., 2015; Palmieri, 2017; Ushioda, 2017; Dörnyei, 2019). Recent studies suggest that motivation for LOTEs is not universal but sensitive to specific LOTEs and social contexts (Ushioda, 2016; Dörnyei and al-Hoorie, 2017; Fraschini and Caruso, 2019; Huang, 2019), highlighting the necessity to explore learners’ motivation for specific LOTEs. While some studies on LOTEs have examined learner motivation in LOTEs such as German and Mandarin (as cited in Huang, 2019), there has been a dearth of research on learning Korean as a LOTE (Mendoza and Phung, 2019; Guo et al., 2021). However, with the emergence of Korea as a geopolitically and economically strong country, as well as an important exporter of its cultural products, there has been an increasing number of Korean language learners both in Anglophone countries (Fraschini and Caruso, 2019) and in Asia (Ushioda, 2017). Accordingly, the extension of studies of LOTEs to Korean learning will not only expand the research scope of LOTE learning, but also will contribute to the comprehensive understanding of language learner motivation.

L2 Motivational Self System (L2MSS) is a comprehensive theory developed by Dörnyei and his colleagues to explore language learners’ motivation with extensive emphasis on self-based perspectives, providing researchers with a theoretical framework for analyzing the complex nature of language learning motivation (MacIntyre, 2010; Xie, 2014; Ushioda, 2016; Dörnyei and al-Hoorie, 2017; Henry, 2017; Dörnyei, 2019). This L2MSS theory, which views learner behavior deriving from a desired future self, comprises three prominent components of motivation: the *ideal L2 self,* the *ought-to L2 self,* and *L2 learning experience.* While the *ideal L2 self* refers to what a language learner would like to become with regard to uses of the target language, the *ought-to L2 self* concerns the beliefs held by learners on what a language learner ought to do in order to meet social and institutional expectations. The third component of *L2 learning experience* refers to “executive motives related to the immediate learning environment and experience” (Dörnyei, 2009, p. 29) in which learners are exposed to the whole range of teacher instruction, the curriculum, peer interaction and so on (Dörnyei, 2009, 2019). While the *ideal L2 self* and the *ought-to L2 self* are considered as future self-guides that trigger L2 learner behavior, other factors have been hypothesized to exert some effect on the two constructs of the *ideal L2 self* and *ought-to L2 self* (e.g., Taguchi et al., 2009; Sugita McEown et al., 2017; Shih and Chang, 2018; etc.). These factors include *family influence*, *instrumentality promotion*, *instrumentality prevention*, a*ttitude to learning L2*, *attitude toward community*, *cultural interest*, and *integrativeness*. According to Dörnyei (2009), motivation to learn L2 also exhibits group characteristics as it is influenced by different social factors and the ongoing context (Henry, 2019). Some longitudinal studies have shown L2 learning motivation fluctuates as learning proceeds in the Chinese schooling context (e.g., Xu and Gao, 2014).

To sum up, given the scarcity of the study in motivation to learn Korean and the complex roles and interactions among affective variables including the L2MSS related variables, this study aims to investigate the Chinese undergraduate students’ motivation to learn Korean, to identify the factors that differentiate between the first-year and the second-year Korean language learners, and to examine the relationships between these motivational factors and the *ideal L2 self* and the *ought-to L2 self* together by investigating the motivation of the Korean language learners in a Sino-Korean joint education program at a Chinese university. In particular, with the application of the logistic regression, the factors that distinguished between the first-year and the second-year Chinese learners of Korean will be identified. Then through canonical correlation analysis, the shared variance between the future self-guides (i.e., the combination of *ideal self* and *ought-to self*) and other factors mentioned above (i.e., *family influence*, *instrumentality promotion*, *instrumentality prevention*, a*ttitude to learning L2*, *attitude toward community*, *cultural interest*, and *integrativeness*) would be examined.

## Literature review

### L2 motivation, L2MSS and motivation for learning LOTEs

Motivation is considered to be one of the most significant constructs for successful L2 learning (MacIntyre, 2010; Boo et al., 2015; Lanvers, 2016; Ushioda, 2016). L2 learner motivation has been widely studied using Gardner and his colleagues’ socio-psychological model and the Socio-Educational Model of L2 Acquisition (Ushioda, 2006, 2016; MacIntyre, 2010; Dörnyei, 2019). In this trend, two factors *integrativeness* and *instrumentality* have been highlighted. *Integrativeness* refers to L2 learners’ willingness to integrate into the target language community, while *instrumentality* refers to L2 learners’ desire to gain positive rewards including college admission and job promotion by learning and using L2. This model has substantially affected L2 research because of its new perspectives on studying L2 motivations that reflect “social, cognitive and affective forces” (MacIntyre, 2010, p. 375) that are important to human beings. However, some have argued that this model does not fully explain what motivates individuals to learn additional languages, as it primarily accounts for English learning in a bilingual context in Canada, and thus making it less applicable for motivation to study L2 in countries where target languages and communities are less accessible (Ryan, 2009; Ushioda, 2017; Dörnyei, 2019).

Dörnyei and his colleagues modified Gardner’s model by incorporating a set of theories in psychology, which included possible selves theory of Markus and Nurius, self-discrepancy theory of Higgins and self-determination theory of Noel (Dörnyei and Ryan, 2015). In this model, three factors are highlighted: the *ideal L2 self*, the *ought-to L2 self*, and *L2 learning experience*. While the *ideal L2 self* represents the aspirations and qualities an L2 learner hopes to demonstrate by using the specific language, the *ought-to L2 self* represents what an L2 learner feels obliged to do by learning and using the target language to avoid potential adverse effects of not learning the particular language (Dörnyei, 2009). *L2 learning experience* concerns how an L2 learner’s mindset is affected by the current learning context, which has recently been interpreted as one’s language learning engagement (Dörnyei, 2009, 2019). Dörnyei’s (2009) L2MSS theory views L2 learning motivation from a self-perspective. Other factors are reconsidered and regarded correlated with these self-based variables in this model. For example, *integrativeness* in Gardner’s model is reconceptualized as language learners’ own attitude or affection for L2 community members (Dörnyei, 2009); in some studies *instrumentality* is further developed into two subcategories: *instrumentality promotion* and *instrumentality prevention* (Taguchi et al., 2009; Huang, 2019). While *instrumentality promotion* is associated with L2 learners’ idealized self-image, such as aspirations for career promotions; *instrumentality prevention* is connected to the motivation to avoid unexpected events, such as exam failures. Thus, *instrumentality prevention* is believed to be linked with one’s *ought-to L2 self*. By focusing on individual’s self-conceptions, L2MSS shifts the criteria of L2 motivation from external convergence to positive end-states within the individual (Dörnyei, 2009; Ushioda, 2017).

The L2MSS theory considers the discrepancy between ones’ perception of the current self and the desired L2 self in the future as an agency that can elicit learners’ cognitive planning and behavioral action (Dörnyei and al-Hoorie, 2017; Dörnyei, 2019). Thus, the *ideal L2 self* and the *ought-to L2 self*, particularly the *ideal L2 self*, are viewed as future self-guides that motivate the learner’s positive behavior and thus push the current self to move closer to the perceived future selves (Dörnyei, 2009). After it has been developed, L2MSS was studied to examine its empirical validity in predicting intended learning efforts in different contexts (e.g., Taguchi et al., 2009; Kormos et al., 2011; Huang et al., 2015; Moskovsky et al., 2016; You and Dörnyei, 2016; Kong et al., 2018; etc.). For example, it had been applied in various contexts to investigate learner motivation for Global English (Taguchi et al., 2009; Kormos et al., 2011; You and Dörnyei, 2016, etc.). The research results demonstrate that the self-based variables were important predictors of intended efforts for Global English (e.g., Csizér and Kormos, 2009; Taguchi et al., 2009; Papi, 2010; Kormos et al., 2011; You and Dörnyei, 2016). The capacity to explain the motivation for L2 learning in different contexts reveals that L2MSS is a highly explanatory theory. L2MSS had also been utilized to predict learner motivation for LOTEs (Oakes, 2013; Sugita McEown et al., 2017; Huang, 2019; etc.), which would be further discussed in the following section.

Despite the applicability of L2MSS, the increased multilingualism (Douglas Fir Group, 2016) together with the paucity of research on LOTEs necessitates a better understanding of LOTE motivation (Boo et al., 2015; Ushioda, 2017; Howard and Oakes, 2021). LOTE studies have been rooted in the field of L2 study, with the explicit goal of describing how a learner builds “a new language system” (as cited in Ushioda, 2017, p. 475). This view was supported by Henry (2010, p. 159) and Dörnyei and Al-Hoorie (2017, p. 458), arguing that LOTE learning can “take the study of English learning as a reference yardstick.” However, Dörnyei and Al-Hoorie (2017) also note that LOTE motivation has specific attributes, with sensitivity to specific language and contexts being the primary concern. Meanwhile, a recent literature reported that L2MSS had been used mainly to study learning motivation for Western European languages and Mandarin, resulting in a dearth of studies on other LOTEs, such as Korean (Mendoza and Phung, 2019).

As pointed out in the aforementioned review, learning Korean as a LOTE is gaining its popularity alongside the growth of Korea’s geopolitical and economic influence (Inhye, 2018; Fraschini and Caruso, 2019). However, research on Korean learning is limited compared to that of other languages (Mendoza and Phung, 2019). Among the few studies available, studies conducted in America and Australia had highlighted learners’ aspiration to integrate into Korean-speaking communities and their interests in Korean cultural products (Lee, 2002; Fraschini and Caruso, 2019). In Korea, a study investigated the motivational tendency of an English language teacher from an English-speaking country to study Korean. The research results indicated that an *ideal L2 self* should be stimulated and elevated by vivid vision of fluent utilization of the Korean language (Gearing and Roger, 2018). Few studies have been done in China to know Korean learners’ motivation. For example, a study on those who studied Korean as their university major showed that the *ideal L2 self* had positive correlation with Korean learning effort, and that learners’ affection for Korean culture stimulated their willingness to integrate into Korean community (Gao, 2010). Given the scarcity of studies on Korean learning and the considerable number of Chinese undergraduate students studying Korean, this study aims to investigate Korean learning motivation of Chinese undergraduate students using L2MSS, describing the levels of motivational variables, i.e., the *ideal L2 self*, the *ought-to L2 self, family influence*, *instrumentality promotion*, *instrumentality prevention*, *attitude to learning Korean*, *cultural interest*, *attitude toward community*, and *integrativeness* to learn Korean.

L2 learners’ motivation is time and context sensitive, implying that it changes as learning progresses (Campbell and Storch, 2011; Busse and Walter, 2013; Xu and Gao, 2014; Henry, 2017; Gearing and Roger, 2018). As a complex and multifaceted construct, L2 motivation can be influenced by context and L2 learning experience, and it could take on distinct characteristics at different learning phases. A longitudinal study conducted in Hungary found that learners maintained their motivation to learn English over several years, while their learning motivation for other languages decreased (Dörnyei and Csizér, 2002). Thus, to better understand the motivational factors associated with the undergraduate Korean learners in different grades, this study aims to identify what variables, i.e., the *ideal L2 self*, the *ought-to L2 self*, *family influence*, *instrumentality promotion*, *instrumentality prevention*, *attitude to Korean learning*, *attitude toward community*, *cultural interes*t and *integrativeness*, could distinguish first-year and second-year Chinese learners of Korean.

### The *ideal L2 self* and the *ought-to L2 self* as future self-guides, and other motivational factors

The positive images of future selves are believed to be able to activate language learners’ cognitive activities and behaviors in L2MSS (Dörnyei, 2009). The *ideal L2 self* and the *ought-to L2 self* act as two self-guides that play important roles in eliciting learner effort. While the *ideal L2 self* has been discovered to be a significant predictor of L2 intended effort (Csizér and Kormos, 2009; Taguchi et al., 2009; Kormos et al., 2011; Busse, 2013; Kim and Kim, 2014; Xie, 2014), the effect of the *ought-to L2 self* on L2 learning effort has been reported as either insignificant (Kormos et al., 2011) or minimal (Csizér and Kormos, 2009; Papi, 2010; Kong et al., 2018). As L2 motivation research continues to progress, it is suggested by researchers that the *ought-to L2 self* deserves more attention in motivation research, particularly in Confucian-influenced societies where the requirements of family and society are highly valued by language learners (Taguchi et al., 2009; Dörnyei and Chan, 2013; Huang et al., 2015).

The predictive power of these two self-guides for intended L2 effort may vary depending on the specific language being studied and the learning context (Huang et al., 2015; Kong et al., 2018; Huang, 2019). Some researchers have investigated the extent to which the *ideal L2 self* and the *ought-to L2 self* are related to motivation for English and LOTEs. For example, some findings indicated that the *ideal L2 self* was predictive of intended effort to learn French, German, and Korean (Csizér and Dörnyei, 2005; Huang et al., 2015). However, the effect of the *ought-to L2 self* on intended L2 effort varies across languages and contexts. For instance, while studies conducted in Europe and South America found that the *ought-to L2 self* did not affect the intended effort to learn German (Oakes, 2013) or English (Kormos et al., 2011), a survey of undergraduate students conducted in Taiwan showed that the *ought-to L2 self* was a significant predictor of learner motivation for English, Japanese, and German, but not for French or Korean (Huang et al., 2015). One of the assumed reasons why *ought-to L2 self* is important for Chinese L2 learners is that they feel obligated to learn a second language to guarantee a higher social status or salary to support their aging family members (Taguchi et al., 2009). Therefore, the *ought-to L2 self* is also considered as an important guide for language learners in China. These findings highlight the need to consider the inclusion of the *ought-to L2 self* as a future self-guide when investigating the learning motivation for a specific language, in particular in Confucian influenced societies (Taguchi et al., 2009; Kormos et al., 2011; Huang et al., 2015).

In literature, it has been found that the future self-guides (i.e., the *ideal L2 self* and the *ought-to L2 self*) are related with other motivational factors (Taguchi et al., 2009; Kormos et al., 2011; Huang et al., 2015; Shih and Chang, 2018). Examples include the simple correlations between *instrumentality promotion* and the *ideal L2 self* (Taguchi et al., 2009), *instrumentality prevention* and the *ought-to self* (Taguchi et al., 2009), and the complex correlation between *attitude to L2 learning* and the *ideal L2 self* and L2 learning motivation (Taguchi et al., 2009; You and Dörnyei, 2016), as well as *family influence* and the *ideal L2 self*, the *ought-to L2 self* and *L2 learning experience* (Kormos et al., 2011; Shih and Chang, 2018). However, few studies have examined the correlation between the set of the *ideal L2 self* and the *ought-to L2 self* and the other set of *family influence*, *instrumentality promotion*, *instrumentality prevention*, *attitude toward learning Korean*, *cultural interest*, *attitude toward community*, and *integrativeness* using canonical correlation, a multivariate analysis. In this technique, the correlation between the independent variable set and the dependent variable set is recognized in investigating simultaneously the correlation between the two sets.

Thus, to understand Chinese undergraduates’ motivation for learning Korean, this study aims to identify factors that distinguish between Korean language learners of different grades, and to examine the relationship between other motivational factors and Korean learners’ future self-guides (i.e., the *ideal L2 self* and the *ought-to L2 self*).

The current study seeks to answer the following three questions:

1. What are levels of the Chinese learners’ ideal L2 self, the ought-to L2 self, family influence, instrumentality promotion, instrumentality prevention, attitude to learning Korean, cultural interest, attitude toward community, and integrativeness to learn Korean as a LOTE?
2. Among the variables listed above, what variables distinguish between first-year and second-year Korean language learners?
3. To what extent may the Korean learners’ ideal L2 self and ought-to self be explained by other motivational variables, i.e., family influence, instrumentality promotion, instrumentality prevention, attitude to learning Korean, cultural interest, attitude toward community, and integrativeness?

## Method

### Participants

The sample consisted of 123 undergraduate students who studied Korean as a part of degree requirement at a university located in eastern China. All students in the sample were enrolled in a Sino-Korean university joint training program, and they were required to study the Korean language for the first two years. The students, 50 males and 72 females (one student’s gender unspecified), were beginner learners of Korean. At the time of this current survey, 66 freshmen had been learning Korean for almost three months, and 57 sophomores had been studying Korean for nearly fifteen months. The students had been attending two Korean language courses: Korean reading and basic Korean communication. After two years of learning Korean, students would have the option to either complete the final two years of their degree at a university in Korea or finish their studies at the Chinese university where they were enrolled currently.

### Instrument

The six-point Likert-type questionnaire surveyed the subjects’ motivation to learn Korean as a LOTE. The 48 items were adapted from the questionnaire used in the investigation of Taguchi et al. (2009), and were translated into Mandarin Chinese by the first author of this study. Three Chinese undergraduate students were asked to review the Chinese version and check the accuracy of the translation. The constructs in the questionnaire are listed in Table 1.

**Table 1:** Introduction and compilation of variables.

Constructs	Brief description	Items	Number of items
Criterion measures	The effort invested in learning Korean	6	2, 10, 17, 23, 27, 31
Ideal self	What one would like to be with the use of Korean	5	5, 11, 21, 28, 32
Ought-to self	What one perceives he ought to be with the use of Korean	6	4, 9, 14, 19, 26, 34
Family influence	Parental support for learning Korean	4	1, 8, 15, 22
Instrumentality (promotion)	Perception of the positive roles of Korean learning or using plays in the future	8	3, 7, 12, 16, 20, 25, 29, 33
Instrumentality (prevention)	Perception of obligations or duties when learning Korean	5	6, 13, 18, 24, 30
Attitude to learning Korean	The “specific situational motives” which relate to learning environment	4	35, 49, 43, 47
Cultural interest	Interest of learners in Korean cultural products	4	37, 41, 45, 46
Attitude toward Korean community	Attitude of learners toward the Korean people and community	3	38, 42, 48
Integrativeness	Aspiration to integrate into the Korean community	3	36, 40, 44

### Data collection and analysis

The survey was conducted in six classes during the last week of the fall semester, 2021. First, an explanation of the study was made to the Korean learners by the first author during their class break, and subsequently the research assistants handed out the hard copies of the questionnaire to the students who were willing to take part in this survey. The samples were collected anonymously, and 143 copies of the survey were returned. Of these, 123 copies with complete information were considered valid. The questionnaire asked students to provide background information about themselves (i.e., gender, grade, and how long they spend in learning Korean after class every week). After the survey was completed, the data were submitted to SPSS for descriptive and inferential statistics. While descriptive statistics of means and standard deviations were calculated to inquire the level of all constructs, Pearson’s correlation coefficient was calculated for correlations between variables. Then, logistic regression analysis was performed to identify the variables that distinguished between first-year and second-year Korean learners. Finally, canonical correlation analysis was employed to examine the relationship between the set of the *ideal L2 self* and the *ought-to L2* (i.e., the future self-guides) and the set of other affective variables (i.e., *family influence*, *instrumentality promotion*, i*nstrumentality prevention*, *attitude to learning Korean*, *cultural interest*, *attitude toward community*, and *integrativeness*).

### Reliability statistics

The reliability of the entire questionnaire as well as each motivational scale was checked. The Cronbach’s Alpha for the whole questionnaire of the 48 items was 0.951, which indicated a high internal consistency. The reliability coefficient for each construct is shown in Table 2.

**Table 2:** Cronbach’s Alpha coefficient and descriptive statistics of the variables.

Variables	Cronbach’s alpha	Descriptive statistics				
		Minimum	Maximum	Mean	Std. Deviation	Variance
Criterion measures	0.898	1.00	6.00	3.9715	1.02867	1.058
Ideal self	0.921	1.00	6.00	4.0911	1.12767	1.272
Ought-to self	0.84	1.17	6.00	3.6260	0.98471	0.970
Family influence	0.834	1.25	6.00	4.1687	0.97115	0.943
Instrumentality promotion	0.925	1.00	6.00	4.1545	0.99397	0.988
Instrumentality prevention	0.806	1.20	6.00	4.5610	0.86561	0.749
Attitude toward learning Korean	0.932	1.00	6.00	4.1748	1.18085	1.394
Cultural interest	0.883	1.00	6.00	4.1138	1.19693	1.433
Attitude toward community	0.863	1.00	6.00	3.8889	1.20840	1.460
Integrativeness	0.812	1.00	6.00	3.8862	1.15220	1.328

## Results

Table 2 displays descriptive statistics of the variables. The subjects of this study had highest mean scores in the categories of *instrumentality prevention* (4.56), *attitude to learning Korean* (4.17), *family influence* (4.17), *instrumental promotion* (4.15), *cultural interest* (4.11), and the *ideal L2 self* (4.09).

As is shown in Table 3, high correlations were observed between *criterion measures* and *instrumentality promotion* (*r* = 0.88), *criterion measures* and *family influence* (r = 0.82), *criterion measures* and the *ideal L2 self* (*r* = 0.811), as well as *criterion measures* and *attitude toward Korean learning* (*r* = 0.80). The weakest correlation was found between *cultural interest* and *instrumental prevention* (*r* = 0.344). While most correlations ranged between 0.50 and 0.70, there was no concern for multicollinearity based on the correlations among variables.

**Table 3:** Pearson correlation coefficients between the variables.

	Criterion measures	Ideal self	Ought-to self	Family influence	Instrumentality (promotion)	Instrumentality (prevention)	Attitude to learning Korean	Cultural interest	Attitude toward community	Integra-tiveness
Criterion measures	1									
Ideal self	0.811^**^	1								
Ought-to self	0.744**	0.612**	1							
Family influence	0.821**	0.731**	0.727**	1						
Instrumentality (promotion)	0.877**	0.832**	0.773**	0.825**	1					
Instrumentality (prevention)	0.585**	0.424**	0.672**	0.529**	0.551**	1				
Attitude to learning Korean	0.795**	0.723**	0.665**	0.726**	0.777**	0.493**	1			
Cultural interest	0.531**	0.580**	0.526**	0.582**	0.580**	0.344**	0.651**	1		
Attitude toward community	0.612**	0.615**	0.627**	0.619**	0.637**	0.471**	0.710**	0.815**	1	
Integrativeness	0.675**	0.654**	0.691**	0.684**	0.676**	0.513**	0.757**	0.737**	0.877**	1

**. Correlation is significant at the 0.01 level (2-tailed).

### Logistic regression analysis

Logistic regression analysis was performed to investigate which variables distinguished first-year Korean learners from the second-year learners. Table 4 shows that the results of the logistic regression analysis were statistically significant (Chi-square = 30.131, df = 10, Sig = *p* < 0.001). Nagelkerke’s R square was 0.297, indicating that almost 30% of the variance the group membership (i.e., the first-year or the second-year Korean language learners) can be explained by the linear combination of the independent variables (see Table 5). The classification Table 6 shows that the logistic regression model classified 75% of subjects correctly.

**Table 4:** Omnibus Tests of Model Coefficients.

		Chi-square	df	Sig.
Step 1	Step	30.131	10	0.001
	Block	30.131	10	0.001
	Model	30.131	10	0.001

**Table 5:** Model Summary.

Step 1	-2 Log likelihood	Cox & Snell R square	Nagelkerke R square
	135.022^a^	0.222	0.297

^a^Estimation terminated at iteration number 5 because parameter estimates changed by less than 0.001.

**Table 6:** Classification Table^a^.

	Observed		Predicted		
			Year		Percentage correct
			0.00	1.00	
Step 1	Year	0.00	50	16	75.8
		1.00	14	40	74.1
	Overall Percentage				75.0

^a^The cut value is 0.500.

The results displayed in Table 7 indicate that *family influence*, *attitude to learning Korean*, and *cultural interests* were statistically significant variables. For every one unit increase in *family influence*, the odds of a student belonging to the second-year group increased by 207.6%. Also, for every one unit increase in *cultural interest*, the odds of a student belonging to the second-year group increased by 165%. However, for every one unit increase in *attitude to learning Korean*, the odds of a student belonging to the second-year group decreased by 25.4%.

**Table 7:** Coefficients of variables in the logistic regression equation.

Variables	B	S.E.	Wald	Df	Sig.	Exp(B)
Criterion measures	0.032	0.538	0.003	1	0.953	1.032
Ideal self	−0.593	0.414	2.052	1	0.152	0.553
Ought-to self	0.092	0.430	0.045	1	0.831	1.096
Family influence	1.124	0.493	5.198	1	0.023	3.076
Instrumentality (pro)	−0.982	0.607	2.621	1	0.105	0.374
Instrumentality (pre)	0.158	0.378	0.174	1	0.677	1.171
Attitude to learning Korean	−1.038	0.420	6.111	1	0.013	0.354
Cultural interest	0.975	0.419	5.401	1	0.020	2.650
Attitude toward community	−0.027	0.471	0.003	1	0.954	0.973
Integrativeness	0.210	0.445	0.223	1	0.637	1.234
Constant	0.009	1.274	0.000	1	0.994	1.009

### Canonical correlation analysis

Canonical correlation analysis was used to answer our third research question: to what extent can the *ideal L2 self* and the *ought-to L2 self* be explained by other variables, such as *family influence*, *instrumentality promotion*, *instrumentality prevention*, *attitude to learning Korean*, *cultural interest*, *attitude toward community*, and *integrativeness*?

The results of the canonical model analysis (Table 8) were statistically significant (Wilks = 0.1226, *p* < 0.001). Both of the canonical roots were also statistically significant. However, when the redundancy was calculated, the second root had the redundancy of only 0.032. Consequently, only the first canonical root was used for the interpretation of the results. The redundancy of the first canonical root was 0.687, which was calculated by multiplying the PV value of 0.806 by 0.853, the squared canonical correlation coefficient. Accordingly, the two sets of variables shared 68.7 percent of variance together; in other words, 68.7% of variance in the set of the *ideal L2 self* and the *ought-to L2 self* was explained by the linear combination of *family influence, instrumental promotion*, *instrumental prevention*, *attitude toward learning Korean*, *cultural interest*, *attitude toward community*, and *integrativeness*.

**Table 8:** Wilks’ Lambda test of significance, squared correlation coefficients and PV value.

	Wilks’ Lambda	Sig.	Sq cor	PV DEP
1	0.1226	0.000	0.853	0.806
2	0.8342	0.002	0.166	0.194

**Figure 1:**
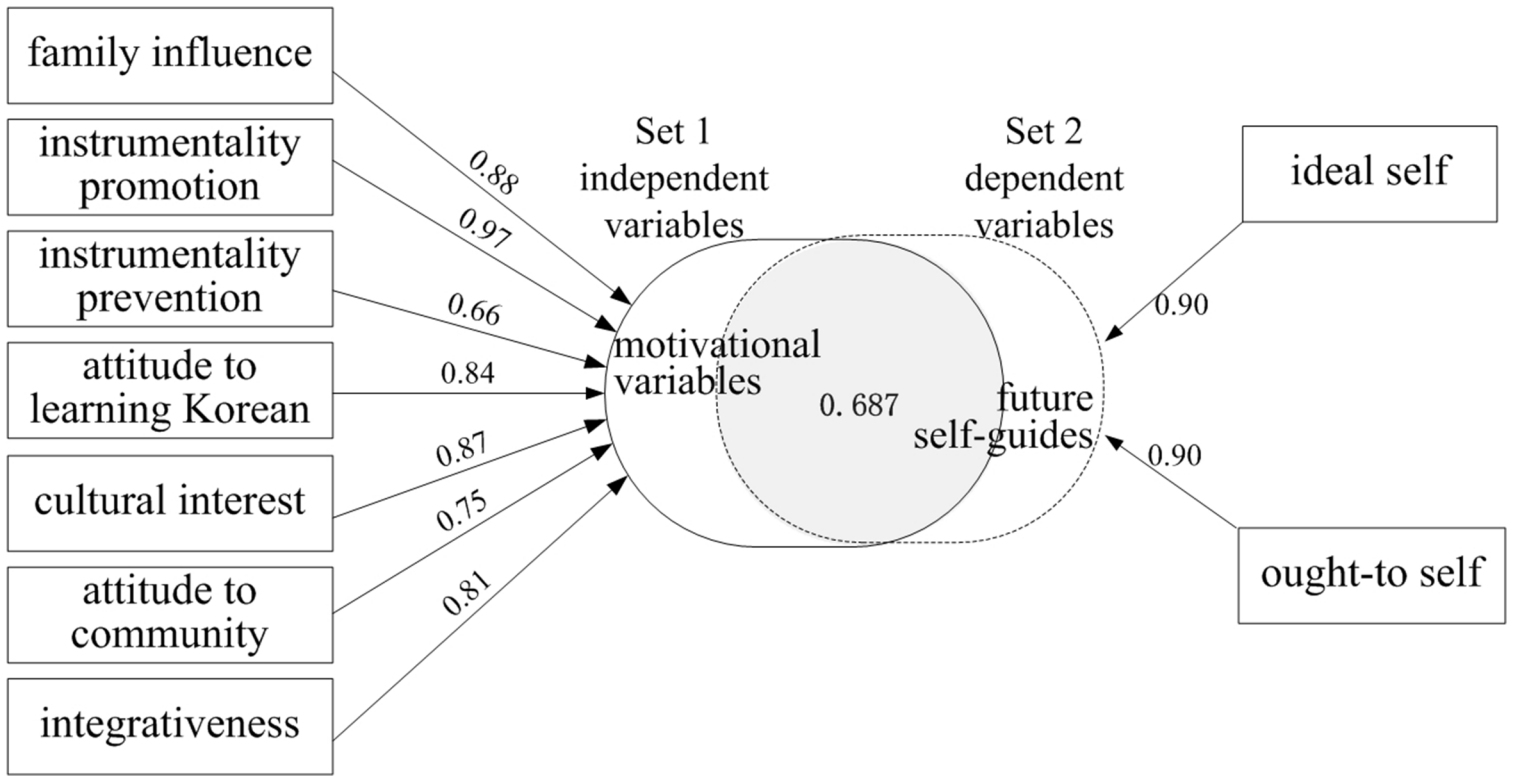
Canonical correlation between Motivational variables (family influence, instrumentality promotion, instrumentality prevention, attitude to learning Korean, cultural interest, attitude toward community, integrativeness) and future self-guides (the ideal L2 self and the ought-to L2 self).

Figure 1 shows the structure coefficients of each set. The structure coefficients of set 1 indicated that its variables (i.e., *family influence*, *instrumentality promotion*, *instrumentality prevention*, *attitude toward learning Korean*, *culture interest*, *attitude to community*, and *integrativeness*) were highly correlated to the independent variate of set 1. Thus, set 1 was named “motivational variables”. Similarly, the structure coefficients in set 2 suggested that the two variables of the *ideal self* and the *ought-to self* were highly correlated to dependent variate of set 2. Based on these structural coefficients, set 2 was named “future self-guides”. With the canonical correlation was factored in, the redundancy statistics was 0.687 or 68.7% of shared variance between the independent variable set and dependent variable set.

## Discussions and implications

Descriptive statistics of motivational factors of the subjects’ Korean learning were used to answer the first research question. As shown in Table 2, the mean scores of six variables were above 4 points out of 6 points, indicating an intermediate-to-advanced level of motivation for learning Korean. This result supports Lasagabaster’s (2017) claim that learning a new language requires a great amount of learning effort. Among the six variables, *instrumentality prevention* had the highest mean score among these beginner learners, indicating they were strongly motivated to pass the Korean exams and to avoid the possible negative consequences. The second highest mean score for *attitude to learning Korean* demonstrated its importance among the subjects, and the importance of this construct would be further discussed in the following section. Meanwhile, the high mean score for *instrumentality promotion* among these beginner learners may suggest that the subjects were learning Korean for practical purposes, such as learning Korean as a means to get admitted to a graduate school.

The second research question was answered by employing a logistic regression analysis. As shown in Table 7, *family influence*, *cultural interest*, and *attitude to Korean learning* were significant variables in distinguishing the first-year from the second-year Korean language learners. *Family influence* was the most important variable in predicting the group membership for the second-year group; in other words, the higher score of *family influence* was reported, the more likely the subjects were to belong to the second-year group. The result suggested that parents’ attitude became more appreciated as the subjects’ school year advanced. Also, the statistical significance of *family influence* that reflects parents’ positive attitude toward Korean learning suggests that the second-year group tend to mark items of *family influence* higher than the first-year group, beginning to recognize the saliency of *family influence*. However, despite the statistical significance, it is necessary that the negative effects of *family influence* are considered. Previous studies demonstrated that learners felt burdened by external expectations and were deprived of their freedom (Brehm and Brehm, 2013; Dörnyei and al-Hoorie, 2017; Liu and Thompson, 2018). Accordingly, moderate attitude and expectations from parents may be optimal in keeping learners focused on learning a foreign language.

*Cultural interest* was another motivational construct that separated the two groups. The logistic regression analysis showed that the second-year subjects had a stronger affinity for Korean culture than the first-year subjects. The result indicates that the more the subjects were exposed to Korean language and culture, the higher interest they held in Korea and its various aspects of culture. This finding corroborated with the study by Huang et al. (2015) in which they found the significant role of *cultural interest* in learning Korean. The statistical significance of *cultural interest* in this current study may be due to the fact as the second-year subjects continued to get engaged in learning Korean longer than the first-year group, they became aware of cultural processes and products manifest in conversations with the two teachers who were serving also as cultural informants, textbooks embedded with Korean culture, and authentic materials available on mass media.

*Attitude to learning Korean*, which measures learners’ evaluation of “situation-specific motives related to the immediate learning environment and experience” (Taguchi et al., 2009, p.75), is a complex and complicated variable in the field of L2 learning, as it could exert impact on motivation directly and through the mediating variable of the *ideal L2 self*. The results of the current study showed that attitude of students toward learning Korean waned after months of Korean language learning. Pedagogical and institutional factors that may have some negative impact on their attitude toward Korean learning. As individuals learn a new language, their novelty of learning a new language may taper off over time. However, while engaged in learning, classroom factors such as “personality, commitment, competence, [and] teaching method (Dörnyei and Ushioda, 2011, p. 148)” of teachers may cause decreased affect among language learners, which may influence the subjects’ attitude toward Korean learning negatively (Dörnyei and Ushioda, 2011). When this current study was being conducted, their Korean courses were taught online due to the COVID-19 pandemic. Thus, it is plausible that the online instruction may have limited teachers’ abilities to fully utilize their teaching expertise and induce the subjects’ positive affect and expectations for the course. In addition, ineffective teaching methods and resultant low teacher efficacy altogether may have played some roles in reducing the subjects’ *attitude to learning Korean*. The textbooks used in courses were indicative of a traditional language teaching with heavy focus on excessive grammar rule learning: deductive presentation of grammar rules, intensive grammar exercises, and error corrections, all of which may have negatively influenced their Korean learning (Sakai and Kikuchi, 2009).

To reduce the negative impact of traditional teaching on learning Korean, more task-based activities may be promoted as an alternative to traditional teaching methods. Communicative activities could reduce anxiety of students, increase their language learning confidence (Nunan, 2004). In addition to wider uses of communicative activities, teachers’ sharing their successful learning experience with students could also be promoted to help students understand what to expect in the long run (Magid and Chan, 2012). This suggestion is grounded in the findings of a previous study, which showed that both the *id*e*al L2 self* and the *ought-to L2 self* may effect attitudes toward learning a foreign language (Shih and Chang, 2018). Thus, to attain the level of the subjects’ attitude toward learning Korean, task-based activities together with activities that could enhance their visions of desired future self are encouraged (Dörnyei, 2009; Magid and Chan, 2012; Safdari, 2021).

To investigate the relationship between the dependent variable set of the *ideal L2 self* and the *ought-to self* (i.e., the future self-guides) and the independent variable set of other motivational factors, canonical correlation analysis was performed. The results in Figure 1 show that the set of the *ideal L2 self* and the *ought-to L2 self* (i.e., the future self-guides) was highly correlated with the set of motivational variables of *family influence*, *instrumentality promotion*, *instrumentality prevention*, *attitude to learning Korean*, *cultural interest*, *attitude toward community*, and *integrativeness*. The coefficients revealed that *instrumentality promotion* and *instrumentality prevention* were important in the independent variate. The importance of those two motivational variables held by the beginner learners resonates with claim that learning another language seems to be an “expedient measure” as these subjects want to be accepted by a better university Zheng et al.’s (2019, p. 601). Accordingly, it is crucial to maintain and enhance their Korean language learning motivation (Dörnyei, 2009; Magid and Chan, 2012; Safdari, 2021). The variables of *integrativeness*, *attitude toward community*, and *cultural interest* held by these learners were positively correlated with the *ideal L2 self* and the *ought-to L2 self* (i.e., the future self-guides). This result is also in agreement with a recent study conducted in Australia, which reveals that subjects in this study tend to envision themselves close to Korean culture and Korean people (Fraschini and Caruso, 2019).

The overall canonical correlation study indicates that a wide range of motivational variables need to be considered when measures are taken to activate these two future self-guides (i.e., the *ideal L2 self* and the *ought-to L2 self*) in learning Korean language. Previous studies have reported that learners’ motivation for foreign languages may be maintained and that their linguistic confidence may be improved by visualizing their desired future states (Dörnyei and Ryan, 2015); and that the more vivid and detailed the perceived L2 states are, the more potential there is to evoke favorable L2 learning behavior (Magid and Chan, 2012). Thus, teachers could help Korean language learners create these self-images by encouraging them to narrate or write down their imaged successful L2 selves, set clear goals and develop detailed action plans throughout Korean learning process (Magid and Chan, 2012; Safdari, 2021). To improve the vividness of the learners’ self-images of using Korean, teachers and institutions may consider providing various tasks that allow learners to imagine themselves as active users of Korean. Teachers may also consider utilizing a variety of resources of Korean movies and video clips that are popular among the language learners, with the purpose to show the learners some real-life conversations and facilitate them to improve their communicative skills (Nunan, 2004; Safdari, 2021). By doing so, learners’ visions are hoped to be kept alive, their affect for Korean culture could be retained, and their Korean language proficiency may be improved (Magid and Chan, 2012; Dörnyei and Ryan, 2015).

## Conclusion

The study investigated motivation of Chinese undergraduate students to learn Korean using Dörnyei’s L2 Motivational Self System. The results indicate that some variables examined had an impact on the subjects’ intended effort to learn Korean as a LOTE. *Family influence*, *attitude to learning Korean*, and *cultural interests* were significant variables that distinguished between first-year and second-year Korean learners. In addition, the study found that the *ideal L2 self* and the *ought-to L2* self (i.e., the future self-guides) of Korean learning shared a high variance with other motivational variables of *family influence*, *instrumentality promotion*, *instrumentality prevention*, *cultural interest*, *attitude toward community* and *integrativeness*. Important implications can be drawn from this study for Korean language teachers and learners. To maintain high levels of motivation in Korean language learners, parents’ constant and mild expectation are welcomed to help learners alleviate their external and internal stress, and keep their Korean learning motivation. Additionally, task-based activities that could inspire Korean learners’ self-images of successful languages users are suggested to sustain their Korean learning efforts.

## Data availability statement

The original contributions presented in the study are included in the article/supplementary material, further inquiries can be directed to the corresponding author.

## Ethics statement

Ethical review and approval were not required for the study on human participants in accordance with the local legislation and institutional requirements. All participants provided their written informed consent.

## Author contributions

LS designed the current study and performed data collection, data interpretation, and manuscript drafting. JS supervised the whole project and conducted data analysis. HL edited and improved the original manuscript. All authors contributed to the article and approved the submitted version.

## Conflict of interest

The authors declare that the research was conducted in the absence of any commercial or financial relationships that could be construed as a potential conflict of interest.

## Publisher’s note

All claims expressed in this article are solely those of the authors and do not necessarily represent those of their affiliated organizations, or those of the publisher, the editors and the reviewers. Any product that may be evaluated in this article, or claim that may be made by its manufacturer, is not guaranteed or endorsed by the publisher.
